# Supplementary material for: Collaborative intelligence in AI: Evaluating the performance of a council of AIs on the USMLE
Source: PLOS Digit Health. 2025 Oct 9;4(10):e0000787. doi: 10.1371/journal.pdig.0000787 (PMC12510544; doi:10.1371/journal.pdig.0000787)
Supplement: S2 File — (ZIP) [file pdig.0000787.s002.zip › S2 Data Raw data files containing unprocessed question inputs and LLM outputs including transcripts of deliberations/STEP3_Question-55-1_Type-MC-NJ.docx]

**Facilitator** **[Round 1]:** A 5-week-old infant is brought to the office by his mother for an initial well-child visit. He was born at 37 weeks' gestation via cesarean delivery because of chorioamnionitis. The infant had fetal distress that was documented by fetal monitoring during labor. The mother became jaundiced in the immediate postpartum period and was found to have hepatitis C (HCV) and HIV infection. She is unaware of her hepatitis B (HBV) status but knows that her son received the hepatitis B vaccination on the second day of life. The infant's weight today is 3912 g (8 lb 10 oz); physical examination shows no abnormalities. The mother wants to know what chance her child has of having contracted HIV or HCV infection from her. The risk for vertical transmission of HIV is approximately 20%. The mother is advised and appropriate management is planned for the infant regarding HIV. To determine the risk of HCV transmission, a recently published study of risk factors for perinatal transmission of HCV in 155 mothers coinfected with HIV and HCV is reviewed. Results of the study are shown: Association of Gestational and Infant Factors With Mother-to-Infant HCV Transmission 155 Mothers with HIV and HCV Infection Factor: Gestational Age <37 weeks - HCV Transmission 8.4%, RR 1.0, 95% CI - Factor: Gestational Age ≥37 weeks - HCV Transmission 8.3%, RR 0.99, 95% CI 0.32 to 3.06, p Value 0.99 Factor: No Cesarean Delivery - HCV Transmission 6.0%, RR 1.0, 95% CI - Factor: With Cesarean Delivery - HCV Transmission 13.3%, RR 2.21, 95% CI 0.69 to 7.06, p Value0.24 Factor: No Chorioamnionitis - HCV Transmission 7.0%, RR 1.0, 95% CI - Factor: With Chorioamnionitis - HCV Transmission 33.3%, RR 4.77, 95% CI 0.86 to 26.3, p Value 0.21 Factor: Did not use Fetal Electrode - HCV Transmission 7.0%, RR 1.0, 95% CI — Factor: Used of Fetal Electrode - HCV Transmission 3.3%, RR 0.66, 95% CI 0.09 to 4.89, p Value 0.99 Factor: Infant Not HIV-infected - HCV Transmission 5.4%, RR 1.0 95% CI — Factor: Infant HIV-infected - HCV Transmission 17.1%, RR 3.19, 95% CI 1.14 to 8.93, p Value 0.04 RR=relative risk; CI=confidence interval Based on these data, which of the following is the most appropriate conclusion regarding the five potential risk factors for transmission of HCV? (A) All factors appeared to have an effect on HCV transmission (B) Fetal electrode monitoring was protective against HCV (C) Infant HIV infection was the only significantly associated factor (D) Maternal chorioamnionitis was the most significantly associated factor (E) No factor was significantly associated

**AI_A:** RESTATING THE QUESTION: The mother has HCV and HIV infection, and we are tasked with interpreting the relative risk and p-value from a study done about maternal to infant HCV transmission. The new mother is concerned about the risk of transmission of the infections to her newborn. Factors for HCV transmission considered were the gestational age at birth, mode of delivery, presence of chorioamnionitis, use of fetal electrode during labor, and infant HIV infection. PLANNING: Initially, we should consider the concepts of Relative Risk (RR) and confidence interval (CI). The RR is a measure of the strength of the relationship between an exposure or risk factor and a disease or outcome. A RR of 1 suggests no association, less than 1 suggests a possible protective effect, and more than 1 indicates an increased risk. CIs provide a range for the estimated RR, and if the CI includes 1, it is generally considered non-significant. Also important is the p-value, which indicates statistical significance; a p-value <0.05 generally indicates statistical significance. CLINICAL REASONING: By applying these definitions to the data presented, we can observe that most of the factors studied have RR >1, suggesting an increased risk of HCV transmission with these conditions. However, for the claim to be statistically significant, we need the p-value to be less than 0.05. Surprisingly, only one factor, infant HIV infection, meets this condition (p-value 0.04), making it the only factor significantly associated with an increased risk of HCV transmission from mother to infant. CONCLUDING: Based on the definitions of RR, CI, and what is considered significant in p-value, only one factor, infant HIV infection, has shown a statistically significant correlation to the risk of mother-to-child transmission of HCV. RESPONSE: Therefore, the most appropriate conclusion is that (C) Infant HIV infection was the only significantly associated factor.

**AI_C:** RESTATING THE QUESTION: An HIV and HCV-infected mother is inquiring about the risk of transmission of these infections to her 5-week-old son. The baby was delivered at 37 weeks via cesarean section due to fetal distress and maternal chorioamnionitis. The mother is jaundiced postpartum, indicating liver disease. A study of 155 HIV and HCV co-infected mothers was reviewed to determine the potential risk factors of perinatal HCV transmission. The question is asking to interpret this data and identify which option is most representative of the transmission risk. PLANNING: To assess the question, we need to understand epidemiological measurements like Relative Risk (RR) and Confidence Interval (CI), and P-value. RR is a statistical measurement of the strength of a risk factor's association with a disease outcome. RR less than 1 indicates a reduced risk of the outcome, RR equals one shows no effect, and RR more than 1 means increase risk. The CI represents the range of values within which the actual RR could lie. The smaller the CI, the more certain we are about the RR. The P-value measures the statistical significance of the hypothesis; if the P-value is smaller than 0.05, the results are statistically significant. CLINICAL REASONING: By reviewing the data we can see effects of different factors on HCV transmission. For gestational age, cesarean delivery, chorioamnionitis, and use of a fetal electrode, the RR values are not significantly different from 1 and the P-values are greater than 0.05, so these factors don't seem significantly associated with HCV transmission. The only factor that has RR different from 1 and a P-value less than 0.05 is "Infant HIV-infected" with RR 3.19 and P-value 0.04 indicating a significant association between infant HIV infection and HCV transmission. CONCLUDING: Based on these interpretations, the most relevant factor impacting HCV transmission appears to be whether the infant has HIV infection, as evidenced by the RR of 3.19 and a p-value of 0.04. RESPONSE: Therefore, the most appropriate conclusion is option (C) "Infant HIV infection was the only significantly associated factor".

**AI_B:** The query is about distinguishing among five potential risk factors for the transmission of HCV (Hepatitis C virus) from a mother to her child: gestational age, mode of delivery (cesarean or vaginal), maternal condition (chorioamnionitis), use of fetal electrode and infant's HIV infection status. The data displays the Relative Risk (RR), 95% Confidence Interval (CI), and p-value for each risk factor. The RR represents the probability of a certain outcome (in this case, HCV transmission) based on potential risk factors. The 95% Confidence Interval shows the range within which the RR is statistically valid; if the CI contains 1, there's no difference in risk. A p-value less than 0.05 implies statistical significance, suggesting the risk factor has an effect on the outcome. Assumptions include: 1) All mentioned factors are potential contributors to HCV transmission, 2) The risk factors' potential influence is correctly represented in the study, and 3) The interpretation of the statistical results (RR, CI, p-value) are accurately applied. Should any of these assumptions be incorrect, the response to the inquiry would be influenced. To answer the query, the presented statistical data needs to be evaluated. Based on the clinical data, Gestational age at birth seems to have no significant effect on the transmission, as RR for both categories (<37 and ≥37 weeks) approximates to 1, and the p-values are above the threshold for significance (0.05). Even though Cesarean delivery and maternal chorioamnionitis yield higher RR percentages, their confidence intervals cross 1, hence, tend to be statistically non-significant. Similarly, the use of a fetal electrode isn't significantly associated with transmission due to an RR less than 1 and a non-significant p-value. However, an Infant's HIV infection status showed an RR more than 1 (3.19), with the p-value being less than 0.05 (0.04), denoting a statistically significant relation. Thus, upon evaluation of the alternate assumptions and clinical data interpretation, it appears that the risk factor to prioritize clinically is the Infant's HIV infection status due to statistical and clinical relevance. Therefore, in consideration of the presented evidence, the appropriate conclusion would indicate that the Infant's HIV infection status was the only significantly associated factor (Choice C) with the vertical transmission of HCV. All other factors, even though seemed to influence transmission, did not show statistically significant associations.

**AI_D:** RESTATING THE QUESTION: The mother of a 5-week-old infant, who has both HIV and Hepatitis C (HCV), is concerned about the risk of her child contracting either virus from her. We have been provided with data from a recent study looking at the risk factors for mother-to-infant transmission of HCV, including gestational age, mode of delivery (cesarean or not), presence of chorioamnionitis, use of fetal electrode, and whether the infant is HIV-infected. Based on this data, we are asked to arrive at the most appropriate conclusion regarding the risk factors for HCV transmission. PLANNING: The assumption here is that the study provides accurate and comprehensive information on the factors that affect the transmission of HCV from mother to child. We could question this assumption by thinking of additional variables that might influence transmission, like maternal viral load or infant immune status. However, as these variables weren't included in the study, our clinical reasoning must be limited to the given data. Therefore, the assumption to prioritize is interpreting and analyzing this given data accurately. CLINICAL REASONING: Looking at the data, the relative risk (RR) score identifies the likelihood of HCV transmission given the presence of each factor. If RR equals 1, it indicates that the factor had no effect on the transmission rate. If RR is greater than 1, it suggests increased risk, and if less than 1, it implies reduced risk. Moreover, the presence of a statistical significance (p-value less than 0.05) indicates that these observations weren't due to chance. In this case, though multiple risk factors had RRs different from 1, only Infant HIV infection had a statistically significant p-value (0.04), indicating a genuine link between this factor and HCV transmission. CONCLUDING: By connecting this reasoning, it's clear that while several factors showed varying RRs, implying potential influence on HCV transmission, Infant HIV infection was the only factor associated with a statistically significant change, suggesting that this was the only proven connection seen in this study. RESPONSE: Hence, the most appropriate conclusion regarding the five potential risk factors for transmission of HCV is that 'Infant HIV infection was the only significantly associated factor' (C).

**AI_E:** RESTATING THE QUESTION: The situation involves assessing the risk factors related to the transmission of Hepatitis C Virus (HCV) from a mother to her infant. The mother in question has both HCV and HIV. Various circumstances of the child's birth are put forward as potential risk factors. These factors are gestational age, type of delivery, whether chorioamnionitis was present, use of a fetal electrode, and the infant's HIV status. The given statistics provide the relative risk (RR) and confidence interval (CI) associated with each factor. The relative risk is a statistical term that describes the increased risk of an event occurring in the exposed group compared to the non-exposed group. A relative risk of 1 means there's no difference in risk between the two groups. Anything greater than 1 indicates a higher risk for the exposed group, and anything less than 1 indicates a lower risk for the exposed group. The p-value is a statistical measure that helps scientists determine whether their hypotheses are right or wrong. It means the probability of an observed (or more extreme) result arising only by chance. PLANNING: The assumptions that could be made are that every factor listed could impact HCV transmission. However, it's also possible that not all of these factors equally contribute to liver disease transmission. We need to examine the data provided for each factor to see if any of these alternate assumptions hold true. For a factor to be significantly associated with HCV transmission, its p-value needs to be less than 0.05, and the RR should be significantly different than 1. CLINICAL REASONING: According to the given information: 1. Gestational age does not seem to significantly affect transmission, as shown by the similar rates for both <37 weeks and ≥37 weeks (each with RR around 1 and p-values of 0.99). 2. Although cesarean delivery shows a higher relative risk and HCV transmission rate, its p-value of 0.24 does not suggest a significant association. 3. While chorioamnionitis presents with an elevated relative risk, its p-value is also above 0.05, suggesting this connection is not statistically significant. 4. The use of a fetal electrode surprisingly showed a lower HCV transmission rate and a relative risk less than 1. However, the high p-value of 0.99 negates its significance. 5. Lastly, the infant's HIV infection status displays a relative risk greater than 1 (3.19) and a p-value less than 0.05 (0.04), indicating this factor is significantly associated with mother to child HCV transmission. CONCLUDING: Therefore, in the given context, considering the p-values and relative risks, among all the presented factors, it appears the only significant factor affecting the transmission of HCV from the mother to the infant is the infant being HIV-positive. RESPONSE: So, the most appropriate conclusion regarding the five potential risk factors for transmission of HCV would be that (C) Infant HIV infection was the only significantly associated factor.

**Facilitator** The consensus statement synthesizing the responses of AI_A, AI_B, AI_C, AI_D, and AI_E is as follows: The question at hand involves assessing the risk factors related to the transmission of Hepatitis C Virus (HCV) from a mother, who is co-infected with HIV and HCV, to her infant. Various factors such as gestational age, mode of delivery, presence of chorioamnionitis, use of a fetal electrode, and the infant's HIV status are considered. To interpret the data, we need to understand the concepts of Relative Risk (RR), Confidence Interval (CI), and p-value. RR is a statistical measure that indicates the strength of the association between a risk factor and a disease outcome. A RR of 1 suggests no association, less than 1 suggests a possible protective effect, and more than 1 indicates an increased risk. The CI provides a range for the estimated RR, and if the CI includes 1, it is generally considered non-significant. The p-value measures the statistical significance of the hypothesis; if the p-value is smaller than 0.05, the results are statistically significant. Upon reviewing the data, most of the factors studied have RR >1, suggesting an increased risk of HCV transmission with these conditions. However, for the claim to be statistically significant, we need the p-value to be less than 0.05. Only one factor, infant HIV infection, meets this condition (p-value 0.04), making it the only factor significantly associated with an increased risk of HCV transmission from mother to infant. Therefore, based on the definitions of RR, CI, and what is considered significant in p-value, the most appropriate conclusion regarding the five potential risk factors for transmission of HCV is that 'Infant HIV infection was the only significantly associated factor' (C). All other factors, even though they seemed to influence transmission, did not show statistically significant associations.
